# Hypoxia‐Mediated Downregulation of Exosomal miR‐128‐3p Promotes Lymphangiogenesis via VEGF‐C in Oral Squamous Cell Carcinoma

**DOI:** 10.1155/ijod/3514074

**Published:** 2026-05-20

**Authors:** Tengchao You, Furong Mao, Kesheng Zhang, Xulin Zhang, Xiaohua Hu

**Affiliations:** ^1^ Department of Oral and Maxillofacial Surgery, Affiliated Stomatological Hospital of Zunyi Medical University, Zunyi Medical University, Zunyi, Guizhou, China, zmc.edu.cn; ^2^ Department of Oral and Maxillofacial Surgery, Wuxi Stomatological Hospital, Wuxi, Jiangsu, China

**Keywords:** exosome, lymphangiogenesis, miR-128-3p, oral squamous cell carcinoma, VEGF-C

## Abstract

**Background:**

Cancer cell‐derived exosomal microRNAs (miRNAs) are crucial mediators of intercellular communication within the tumor microenvironment, including with lymphatic endothelial cells (LECs). Hypoxia, a key driver of tumor aggressiveness, modulates exosome release and cargo. However, the specific functions of exosomal miRNAs derived from hypoxic oral squamous cell carcinoma (OSCC) cells are not well defined.

**Methods:**

We employed a two‐phase strategy. In the discovery phase, miRNA sequencing was performed on exosomes from hypoxic exosomes and normoxic exosomes (Norm‐exos) Tca‐8113 cells, and their pro‐lymphangiogenic effects on Human LECs (HLECs) were assessed. In the validation phase, using SCC‐9 cells, we investigated the functional role of a candidate miRNA, miR‐128‐3p, through gain‐of‐function experiments and identified its target.

**Results:**

Hypoxic OSCC‐derived exosomes (Hypo‐exos) were internalized by HLECs and significantly enhanced their proliferation, migration, and tube formation. miR‐128‐3p was markedly downregulated in Hypo‐exos. Overexpression of miR‐128‐3p reversed the pro‐lymphangiogenic effects of Hypo‐exos and reduced vascular endothelial growth factor (VEGF)‐C expression. A dual‐luciferase reporter assay confirmed VEGF‐C as a direct target of miR‐128‐3p.

**Conclusion:**

Hypo‐exos promote lymphangiogenesis, partly through the downregulation of exosomal miR‐128‐3p, which directly targets and inhibits VEGF‐C. This novel miR‐128‐3p/VEGF‐C axis presents a potential therapeutic target for mitigating lymphangiogenesis and metastasis in OSCC.

## 1. Introduction

Oral squamous cell carcinoma (OSCC) accounts for more than 90% of all head and neck malignancies [1]. Characterized by a high incidence of lymphatic vessel formation, OSCC frequently results in lymph node metastasis [2]. While the 5‐year survival rate for localized OSCC surpasses 80%, this figure drops significantly to 40% when lymph nodes are affected, and further decreases to 20% in cases with distant metastasis [3].

Hypoxia, defined as tissue oxygen pressure below 5–10 mmHg, is a critical driver of cancer metastasis [4]. This condition, common in solid tumors due to the imbalance between oxygen demand and supply, accelerates tumor progression by activating specific signaling pathways [5]. It stimulates angiogenesis and lymphangiogenesis, facilitating tumor dissemination to other sites [6]. The hypoxia‐inducible factor (HIF) family regulates cellular adaptations to hypoxia, inducing lymphangiogenesis by controlling the expression of vascular endothelial growth factor (VEGF)‐C. Increased levels of VEGF‐C have been linked to lymph node metastasis, recurrence, and a poorer prognosis in patients with OSCC [7].

Exosomes, a class of extracellular vesicles (EVs) measuring 50–150 nm in diameter, are secreted by various cell types, including dendritic cells, B cells, T cells, mast cells, epithelial cells, and tumor cells. These vesicles transport bioactive molecules such as proteins, lipids, and non‐coding RNAs to recipient cells, serving as mediators of intercellular communication and modulators of signaling pathways [8]. Hypoxia has been shown to increase the release of exosomes and alter their cargo and functionality [9], highlighting their importance in the tumor microenvironment and their role in tumor progression and metastasis [10].

MicroRNAs (miRNAs), which are abundant in tumor‐derived exosomes, play a crucial role in gene regulation within target cells, thereby influencing multiple facets of cancer progression. Studies have indicated that hypoxic conditions can elevate the levels of miR‐21 and miR‐210 in exosomes, leading to enhanced cell migration, invasiveness, and angiogenesis in recipient cells [11, 12]. Collectively, this evidence suggests that exosomes act as vehicles transferring miRNAs from cancer cells to human lymphatic endothelial cells (HLECs). In hypoxic environments, these exosomal miRNAs may modulate the biological activity of HLECs, potentially facilitating tumor dissemination. In recent years, a considerable body of research has highlighted the function of miR‐128 as a tumor suppressor, inhibiting tumor growth and metastasis through multiple pathways, it is closely linked with the development and progression of various malignancies [13]. Furthermore, studies have confirmed that miR‐128‐3p can influence lymphatic metastasis in tumors; however, the underlying mechanisms remain to be fully elucidated [14]. Given the documented downregulation and tumor‐suppressive function of miR‐128‐3p in OSCC, we hypothesized that its specific packaging into exosomes under hypoxia could be a novel mechanism controlling lymphangiogenesis.

To systematically test this, we adopted a two‐phase strategy: (1) a discovery phase using the Tca‐8113 cell line to establish the pro‐lymphangiogenic capacity of hypoxic OSCC‐derived exosomes (Hypo‐exos) and profile their miRNA content and (2) a validation phase using the SCC‐9 cell line to independently confirm the function and molecular target of a candidate miRNA. From the discovery screen, miR‐128‐3p emerged as a lead candidate due to its significant downregulation in Hypo‐exos and its computationally predicted targeting of lymphangiogenic genes, leading us to focus on it for mechanistic validation.

## 2. Materials and Methods

### 2.1. Cell Culture and Hypoxic Exposure

This study utilized OSCC cell lines Tca‐8113 and SCC‐9 (ATCC; CRL‐1629), employing a discovery–validation strategy: initial phenotypic screening and miRNA sequencing were performed using Tca‐8113, while functional validation experiments for the candidate miRNA, miR‐128‐3p, were conducted in SCC‐9 to confirm the broader relevance of the findings. Human embryonic kidney cells 293T (CSTR: 19375.09.3101HUMSCSP502) and HLECs were also used. All cells were cultured at 37°C with 5% CO_2_ in DMEM/F12 (Gibco; MA, USA) medium containing 10% fetal bovine serum (FBS; Gibco, MA, USA) and 1% penicillin–streptomycin (Solarbio; Beijing, China), with HLECs using 5% FBS. For hypoxic treatment, cells were incubated for 24 h in an HGO‐50 cell culture incubator (Cqjiangxue, Chongqing, China) with 1% O_2_, 94% N_2_, and 5% CO_2_, using normoxic conditions (20% O_2_) as controls. Prior to exosome isolation, cells were cultured with medium supplemented with 10% exosome‐depleted FBS, prepared by overnight ultracentrifugation at 100,000 × *g* to remove bovine serum‐derived exosomes [15].

### 2.2. Cell Transfection

The miR‐128‐3p mimic and its corresponding negative control (mimic‐NC) were purchased from Hanbio Biotechnology (Shanghai, China). The OSCC cell line SCC‐9 was transfected with 60 nM of the miR‐128‐3p mimic or mimic‐NC using Lipofectamine 3000 (Invitrogen, USA) following the manufacturer’s protocol to overexpress miR‐128‐3p.

### 2.3. RNA Extraction and Reverse Transcription Quantitative Polymerase Chain Reaction (RT‐qPCR)

Total RNA was extracted from cell and exosome samples using the FreeZol reagent (Vazyme, Jiangsu, China), followed by reverse transcription into cDNA using the miRNA 1st Strand cDNA Synthesis Kit (by stem‐loop) (Vazyme). Subsequently, cDNA synthesis was conducted using the HiScript II Q RT SuperMix for qPCR (+ gDNA wiper) (Vazyme), and RT‐qPCR was performed with the miRNA Universal SYBR qPCR Master Mix (Vazyme). Relative gene expressions were calculated by 2^−ΔΔCt^ method.

A universal reverse primer included in the miRNA 1^st^ strand cDNA synthesis kit was used for all miRNA amplifications. The primers used for the amplification of these miRNAs and the endogenous control U6 were synthesized by GENERAL BIOL (Anhui, China). The forward primer sequences are as follows: miR‐128‐3p: CGCGTCACAGTGAACCGGT; miR‐365b‐3p: CGCGTAATGCCCCTAAAAAT; miR‐132‐3p: GCGCGTAACAGTCTACAGCCA; miR‐21‐3p: GCGCAACACCAGTCGATG; U6 forward: CTCGCTTCGGCAGCACA, U6 reverse: AACGCTTCACGAATTTGCGT.

### 2.4. Isolation and Identification of Exosomes

Exosomes were isolated from the supernatants of OSCC cells using sequential centrifugation steps as previously described [16]. Briefly, the supernatant was centrifuged at 300 × *g* to remove dead cells, followed by 2000 × *g* to eliminate cellular debris, and 10,000 × g to exclude larger vesicles. The resulting supernatant was filtered through a 0.22 μm membrane (Merck, Darmstadt, Germany) and then ultracentrifuged at 100,000 × *g*. The exosome pellet was washed, resuspended in PBS, and stored at −80°C or used immediately.

The concentration of isolated exosomes was determined by measuring the total protein content using a BCA Protein Assay Kit (Beyotime, Shanghai, China), according to the manufacturer’s instructions. For all functional experiments (proliferation, migration, and tube formation assays), HLECs were treated with exosomes at a final concentration of 100 μg/mL for the indicated time periods. This concentration was selected based on preliminary dose‐finding experiments and is consistent with concentrations used in prior literature on exosome‐mediated cellular communication.

For morphological examination, exosomes were visualized by transmission electron microscopy (TEM; Hitachi, Tokyo, Japan). Additionally, nanoparticle tracking analysis (NTA) and nano‐flow cytometry using a Flow NanoAnalyzer (NanoFCM, Fujian, China) were employed to assess size distribution, concentration, and surface marker expression (CD9 and CD81).

### 2.5. Western Blot Analysis

Proteins were extracted with RIPA buffer (Solarbio) and quantified using a BCA kit (Beyotime). Equal amounts of protein were separated by SDS–PAGE and transferred to PVDF membranes (Millipore, MA, USA). Membranes were blocked with 5% skim milk, then incubated with primary antibodies anti‐HIF‐1α (GB111339; Servicebio, Hubei, China), anti‐VEGF‐C (YP‐Ab‐16012; UpingBio, Zhejiang, China) and anti‐β‐actin (GB15003; Servicebio) at 4 °C overnight, followed by a secondary antibody incubation for 1 h at room temperature. β‐actin served as a loading control. Detection was performed using the ChemiDoc MP Imaging system (Bio‐Rad, CA, USA).

### 2.6. Exosome Labeling and Uptake Assays

Purified exosomes were labeled with PKH67 (Umibio, Shanghai, China) by incubation in 500 µL Diluent C with 4 µL PKH67 for 4 min in the dark at room temperature. To remove unincorporated dye and potential aggregates, the reaction mixture was subjected to ultracentrifugation at 100,000 × *g* for 70 min, preceded by a quenching step with BSA in PBS. The resulting pellet of labeled exosomes was resuspended in PBS and used immediately or stored at −20°C. It should be noted that while thorough washing was performed to remove unincorporated dye, a formal dye‐only control was not included in this study.

For uptake assays, HLECs were exposed to the labeled exosomes for 12 h. Cells were then processed for imaging: washed, fixed, permeabilized, and stained with Phalloidin (Solarbio, Beijing, China) for cytoskeleton and DAPI for nuclei. Confocal microscopy (Leica, Wetzlar, Germany) was used to observe and capture images. For quantification, fluorescence images from at least three random fields per group were analyzed using ImageJ software (National Institutes of Health, USA). Mean fluorescence intensity per cell was measured after setting a constant threshold and subtracting the background. Data are presented as relative fluorescence intensity compared to the normoxic exosomes (Norm‐exos; Tca‐8113).

### 2.7. Cell Counting Kit‐8 (CCK‐8)

Cells were seeded at 5 × 10^3^ per well in 96‐well plates. Proliferation was evaluated at 0, 24, 48, and 72 h post‐plating by adding 10 μL of CCK‐8 solution to each well and incubating for 2 h. The OD at 450 nm was then measured using a multifunctional enzyme‐linked analyzer (Thermo Fisher Scientific, MA, USA). The experiment was performed in triplicate.

### 2.8. Colony Formation Assays

After 96 h of culture, HLECs were harvested by trypsinization, centrifuged, and enumerated. The cells were seeded per well in six‐well plates pre‐filled with medium conditioned by tumor‐derived exosomes. After 12 days, colonies were fixed, stained with crystal violet, and rinsed. Colonies of >10 cells were enumerated to assess formation rate. The experiment was performed in triplicate.

### 2.9. Scratch Assays

Cells were reseeded onto 12‐well plates to create a confluent monolayer. The cell monolayer was scratched using a 200 μL pipette tip, then treated with tumor exosomes. At 0, 12, and 24 h post‐scratch, images of the scratch area were captured. The percent wound closure (%) = migrated cell surface area/total surface area × 100.

### 2.10. Transwell Assays

When HLECs reached confluence, they were trypsinized, centrifuged, and counted. Cells (2 × 10^5^) in serum‐free medium were seeded into the upper chamber of a Transwell insert, while 150 μL of exosome suspension was added to the lower chamber. Lower chambers contained 150 μL complete medium with tumor‐derived exosomes. The plate was incubated for 12 h at 37°C in 5% CO_2_. After incubation, the medium in the upper chamber was removed, and the inserts were washed 2–3 times with PBS. Cells were fixed with fixative solution for 30 min, then air‐dried. Crystal violet staining was performed for 20 min, followed by gentle removal of non‐migrated cells on the top surface using a cotton swab. After another 2–3 PBS washes and air‐drying, migrated cells were observed and counted under a 100× microscope from five random fields. The experiment was performed in triplicate.

### 2.11. Tube Formation Assay

Matrigel (BD Biosciences, NJ, USA), diluted 1:1 with chilled culture medium, was added to 96‐well plates (50 μL/well) and solidified at 37°C for 45 min. Next, 8 × 10^4^ HLECs were seeded on top and co‐incubated with tumor‐derived exosomes for 12 h. Capillary‐like structures were imaged using an inverted microscope, and total tube length was quantified via ImageJ. The experiment was performed in triplicate.

### 2.12. miRNA Sequencing and Bioinformatics Analysis

Exosomal total RNA was processed for miRNA library construction and sequencing by LC‐Bio (Zhejiang, China), utilizing TruSeq Small RNA Sample Prep Kits (Illumina, CA, USA). Sequencing was conducted on an Illumina HiSeq 2000/2500 platform. Analysis included adapter trimming, contaminant removal, length selection (18–26 nt), alignment against mRNA, RFam, and Repbase databases, miRNA identification through precursor and genomic database alignment, differential expression analysis, and target gene prediction. Prediction was performed using databases such as miRTarBase, miRDB, Targetscan, and TarBase. Gene Ontology (GO) and Kyoto Encyclopedia of Genes and Genomes (KEGG) pathway annotations were applied to abundant miRNAs and their targets.

### 2.13. Dual Luciferase Reporter Assays

The psiCHECK‐2 vector was digested with XhoI and NotI, purified, and then PCR‐amplified VEGFC 3′UTR WT and MUT fragments were ligated into it. Following transformation into DH5α cells, antibiotic selection, and sequence verification, the resultant plasmids were co‐transfected with hsa‐miR‐128‐3p or a control miRNA into HEK‐293T cells. Luciferase activity was assessed 48 h later. The experiment was performed in triplicate.

### 2.14. Statistical Analysis

Statistical analyses were conducted using SPSS 29.0 (IBM) and GraphPad Prism 9.5 (GraphPad Software). All quantitative experiments were independently repeated at least three times (*n* ≥ 3) to ensure reproducibility. Data from technical replicates within a single experiment were averaged to represent one biological replicate. Data are expressed as mean ± SD. Comparisons between groups were made using Student’s *t*‐test for two samples and one‐way ANOVA for multiple groups. A *p*‐value of less than 0.05 was considered statistically significant.

## 3. Result

### 3.1. Identification of Hypoxic OSCC Cells and Exosomes Derived From OSCC Cells Conditioned Medium

**Figure 1:**
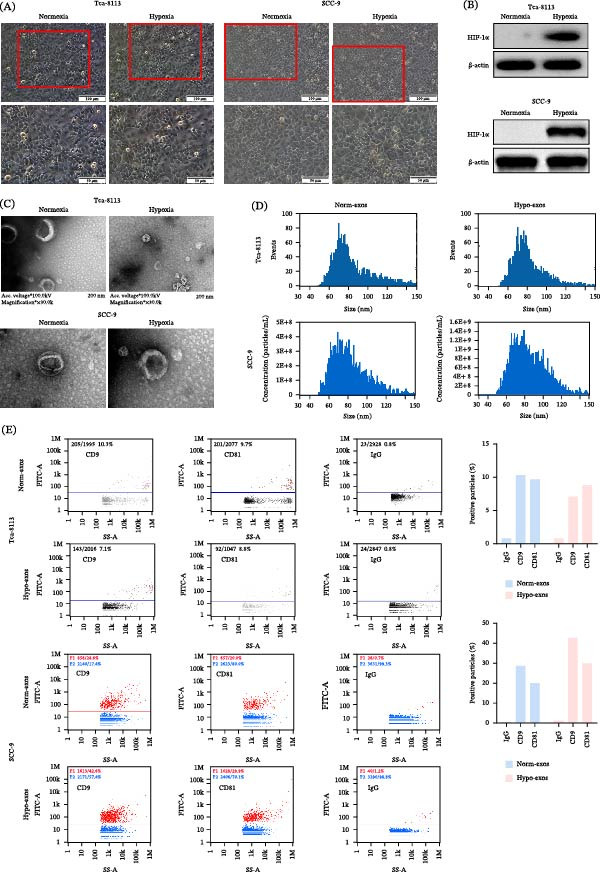
Characterization of exosomes derived from OSCC cells. (A) Cell morphology under normoxic and hypoxic conditions. (B) Expression of HIF‐1α under hypoxic and normoxic conditions. (C) Representative TEM picture of exosomes. (D) NTA was used to determine the particle concentration and size distribution. (E) The expression of exosome surface proteins CD9 and CD81 were detected by nano‐flow cytometry.

To model the tumor microenvironment, OSCC cell lines (Tca‐8113 and SCC‐9) were cultured under hypoxic (1% O_2_) or normoxic (20% O_2_) conditions for 24 h. While cellular morphology remained unchanged (Figure 1A), hypoxic exposure induced a robust upregulation of HIF‐1α protein (Figure 1B), confirming the successful establishment of the hypoxia model.

Exosomes were subsequently isolated from conditioned media via differential ultracentrifugation. TEM revealed cup‐shaped vesicles with a characteristic lipid bilayer (Figure 1C). NTA confirmed that the majority of the particles had a diameter of approximately 80 nm (Figure 1D). Using nano‐flow cytometry, we detected the specific surface markers CD9 and CD81 on both Norm‐exos and hypoxic exosomes (Figure 1E), which were found to be positively expressed. Collectively, these data verify the successful isolation of high‐purity exosomes.

### 3.2. Hypo‐Exos Enhanced the Proliferation, Migration, and Tube Formation Capabilities of HLECs

**Figure 2:**
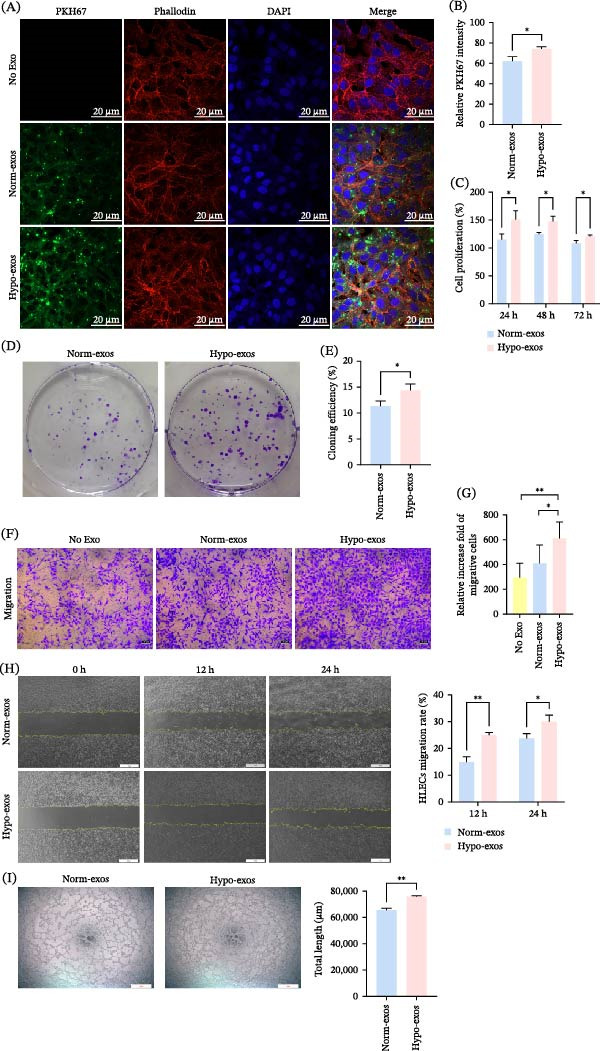
Effects of Hypo‐exos on HLECs. HLECs were treated with exosomes derived from Tca‐8113 cells (Tca‐8113‐exos) at a final concentration of 100 μg/mL for all functional assays. (A) Internalization of PKH67‐labeled Tca‐8113‐exos by HLECs (scale bar, 20 μm). (B) Quantification of the relative fluorescence intensity. (C) CCK‐8 cell proliferation assay in HLECs after treatment with Tca‐8113‐exos. (D) Representative images and (E) quantitative analysis of colony formation assays in HLECs after treatment with Tca‐8113‐exos. (F) Representative images and (G) quantitative analysis of HLEC migration assessed by Transwell assays (scale bar, 50 μm). (H) HLEC migration assessed by scratch wound healing assays at 0, 12, and 24 h (scale bar, 500 μm). (I) The effects of Hypo‐exos (Tca‐8113) on the tube formation detected by tube formation assays (scale bar, 500 μm). All data were mean ± SD from three independent experiments (*n* = 3). ^∗^ *p* < 0.05;  ^∗∗^ *p* < 0.01.

To investigate the biological effects of Hypo‐exos on HLECs, cells were treated with exosomes (at a final concentration of 100 μg/mL) derived under hypoxic conditions, and subsequent phenotypic changes were observed. The Tca‐8113‐exos (Tca‐8113‐derived exosomes) were labeled with fluorescent PKH67, and their internalization by HLECs was confirmed by fluorescent microscopy (Figure 2A). Quantitative analysis of the fluorescence intensity revealed a significant increase in the uptake of Hypo‐exos (Tca‐8113) compared to Norm‐exos (Tca‐8113) (Figure 2B), suggesting that hypoxic preconditioning promotes the delivery of exosomal cargo to HLECs even when applied at equal protein concentrations. The impact of Hypo‐exos (Tca‐8113) on HLEC proliferation was evaluated using CCK‐8 and colony formation assays. The CCK‐8 assay showed that Hypo‐exos (Tca‐8113) significantly enhanced HLEC proliferation over time compared to the Norm‐exos (Tca‐8113) (Figure 2C). This pro‐proliferative effect was further corroborated by a marked increase in colony formation ability, as evidenced by both representative images and quantitative analysis (Figure 2D,E). We next examined the effect of Hypo‐exos (Tca‐8113) on HLEC migration. Transwell migration assays demonstrated a significant increase in migrated cells in the Hypo‐exos (Tca‐8113), with results shown in representative images and their quantification (Figure 2F,G). Consistent with this, scratch wound healing assays also revealed an enhanced migratory capacity of HLECs treated with Hypo‐exos (Tca‐8113) at 12 and 24 h (Figure 2H). Furthermore, tube formation assays indicated that the tubulogenesis potential of HLECs was markedly greater upon treatment with Hypo‐exos (Tca‐8113) compared to Norm‐exos (Tca‐8113) (Figure 2I). In summary, these results demonstrate that Hypo‐exos significantly enhance the proliferation, migration, and tube formation of HLECs in vitro.

### 3.3. miRNA Expression Profile of Hypoxic Tumor‐Derived Exosomes

**Figure 3:**
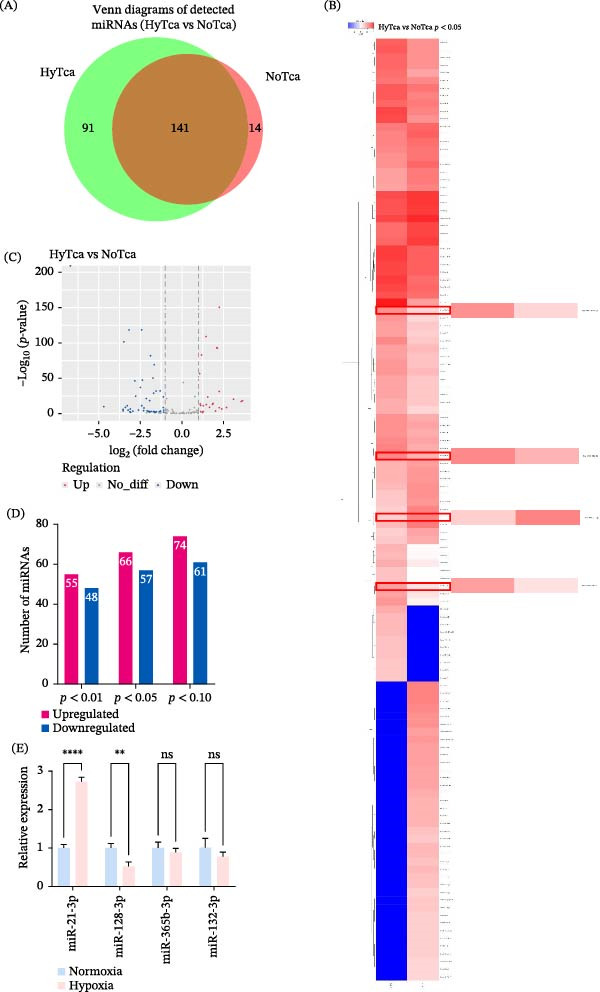
Differential exosomal miRNAs expression. (A) Intersection of differentially expressed exosomal miRNAs. (B) Heatmap of differentially expressed exosomal miRNA sequencing. The positions of the four candidate miRNAs (miR‐128‐3p, miR‐365b‐3p, miR‐21‐3p, and miR‐132‐3p) are explicitly indicated. (C) The volcano map indicates differentially expressed miRNAs from RNA sequencing. (D) Upregulation analysis of differentially expressed miRNAs. (E) Validation of the expression levels of four miRNAs. All data were mean ± SD; the RT‐qPCR data (E) are from three independent experiments (*n* = 3).  ^∗∗^ *p* < 0.01;  ^∗∗∗∗^ *p* < 0.0001; ns, not significant.

Previous research has established that exosomes, which are vesicles enriched with miRNAs, can be internalized by recipient cells, thereby modulating their functional activities [17]. To identify exosomal miRNAs associated with lymphangiogenesis, we conducted miRNA‐seq on total RNA extracted from exosomes of OSCC cell line Tca‐8113 cultured under normoxic and hypoxic conditions. Following a series of quality control measures, the expression levels of miRNA were calculated. 141 miRNAs were simultaneously identified in both groups (Figure 3A). The overlap and unique distribution of miRNAs between Norm‐exos and hypoxic exosomes are visualized in a Venn diagram, while their expression patterns are displayed in a heatmap (Figure 3B) and a volcano plot (Figure 3C).

We next performed a comparative analysis of miRNA expression levels in exosomes derived from normoxic versus hypoxic conditions. Applying a threshold of |log_2_ (fold change)| ≥ 1.5 and a *p*‐value < 0.05, we identified 123 significantly differentially expressed miRNAs. Among these, 66 were upregulated and 57 were downregulated in hypoxic exosomes compared to their normoxic counterparts (Figure 3D). To experimentally validate the sequencing data, we selected four miRNAs—miR‐128‐3p, miR‐365b‐3p, miR‐21‐3p, and miR‐132‐3p—for RT‐qPCR analysis based on their pronounced differential expression (Table 1). Consistent with the miRNA‐seq results, RT‐qPCR analysis of exosomes from the independent OSCC cell line SCC‐9 confirmed a significant upregulation of miR‐21‐3p and a significant downregulation of miR‐128‐3p under hypoxic conditions (Figure 3E).

**Table 1:** Exosome differential miRNAs.

miR_name	Log_2_ (fold_change)	*p*‐Value	Up/down
hsa‐miR‐21‐3p	3.57	9.5000000E − 18	Up
hsa‐miR‐128‐3p	−3.54	1.3099791E − 06	Down
hsa‐miR‐365b‐3p	−3.39	1.7355898E − 09	Down
hsa‐miR‐132‐3p	−2.28	1.8366938E − 08	Down

### 3.4. Differential miRNA Enrichment Analysis

**Figure 4:**
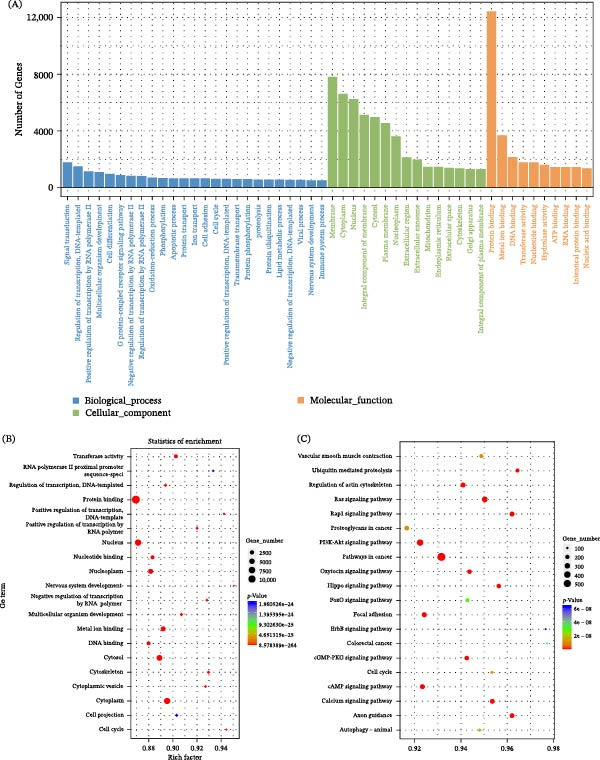
Exosomal differential miRNA enrichment analysis. (A) GO enrichment analysis histogram. (B) GO enrichment analysis bubble diagram. (C) KEGG biological pathway analysis bubble map.

Utilizing databases such as miRWalk, TargetScan, and miRanda, we identified 3802 target genes for 123 differentially expressed miRNAs, setting thresholds at miRWalk_score ≥ 7.0, TargetScan_score ≥ 50, and miranda_Energy < −10. GO analysis revealed that these targets are significantly involved in biological processes like transcription regulation from RNA polymerase II promoters, signal transduction, and multicellular organism development, localized primarily in cell membranes and nuclei (Figure 4A), and exhibit molecular functions related to protein and DNA binding (Figure 4B). KEGG pathway analysis further showed enrichment in pathways, including mTOR signaling, regulation of stem cell pluripotency, pancreatic cancer, and PI3K/AKT signaling, among others (Figure 4C).

### 3.5. Exosomal miR‐128‐3p Inhibits the Proliferation, Migration, and Tube Formation Capabilities of HLECs

**Figure 5:**
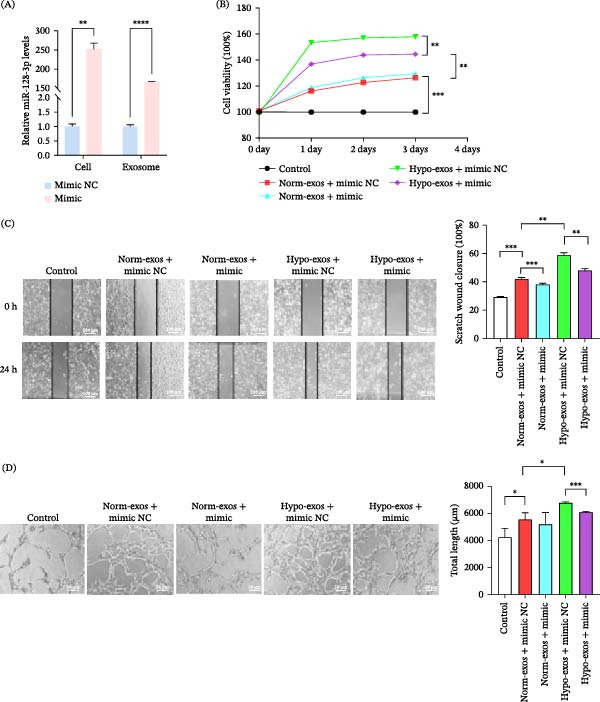
Effects of exosomal miR‐128‐3p on HLECs. (A) RT‐qPCR results of SCC‐9 and SCC‐9‐exos after transfection with miR‐128‐3p mimic. (B–D) The effects of miR‐128‐3p overexpression on the proliferation, migration, and tube formation capabilities of HLECs detected by CCK‐8 cell proliferation assay, scratch assays (scale bar, 100 μm), and tube formation assays (scale bar, 50 μm). All data were mean ± SD from three independent experiments (*n* = 3).  ^∗^ *p* < 0.05;  ^∗∗^ *p* < 0.01;  ^∗∗∗^ *p* < 0.001;  ^∗∗∗∗^ *p* < 0.0001.

We chose miR‐128‐3p for in‐depth investigation due to its significant downregulation in Tca‐8113‐exos under hypoxic conditions. To explore the functional role of exosomal miR‐128‐3p, we overexpressed it using mimic. Transfection with miR‐128‐3p mimic resulted in a marked upregulation of miR‐128‐3p levels in OSCC cell line SCC‐9 and SCC‐9‐exos (SCC‐9‐derived exosomes) (Figure 5A). HLECs were then treated with SCC‐9‐exos cultured under normoxic and hypoxic conditions, originating from SCC‐9 transfected with either a mimic NC or miR‐128‐3p mimic. CCK‐8 for cell proliferation, scratch wound healing for cell migration, and tube formation for angiogenesis (Figure 5B–D), revealed that the addition of SCC‐9‐exos significantly enhanced the proliferation, migration, and tube formation potential of HLECs compared to controls. Hypo‐exos (SCC‐9) displayed a more pronounced stimulatory effect than those from normoxic conditions. However, when miR‐128‐3p mimic was transfected, the stimulatory effects of exosomes on these cellular processes were partially inhibited in HLECs.

### 3.6. Relation Between miR‐128‐3p and VEGF‐C

**Figure 6:**
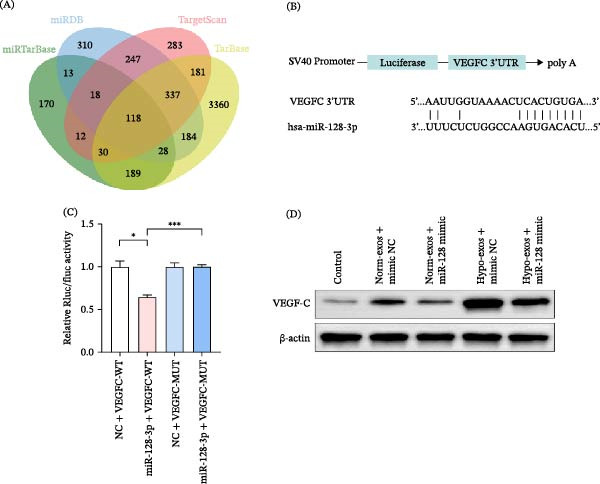
miR‐128‐3p directly targeted VEGF‐C. (A) Venn diagram of the number of predicted targets in four prediction databases (miRTarBase, miRDB, Targetscan, and TarBase). (B) miR‐128‐3p binding Site on VEGF‐C. (C) Luciferase activities of WT or MUT VEGF‐C 3^′^UTR in HEK‐293T cells. (D) Expression of VEGF‐C in HLECs after co‐culturing with SCC‐9‐exos derived from SCC‐9 cells overexpressing miR‐128‐3p under hypoxic or normoxic conditions. The luciferase assay data (C) are presented as mean ± SD from three independent transfections (*n* = 3). All data were mean ± SD;  ^∗^ *p* < 0.05;  ^∗∗∗^ *p* < 0.001.

To identify potential cellular targets of miR‐128‐3p, we conducted an integrative bioinformatic analysis by cross‐referencing data from four established online databases: miRTarBase [18], miRDB [19], TargetScan [20], and miRBase [21] (Figure 6A). The analysis suggested VEGF‐C as the target of miR‐128‐3p (Figure 6B). Co‐transfection of miR‐128‐3p with VEGF‐C and its mutant form in HEK‐293T cells showed regulation of the luciferase reporter, with binding site mutation restoring expression, confirming their interaction and the binding site’s validity (Figure 6C). Consistent with these findings, western blot analysis confirmed a significant decrease in VEGF‐C protein expression in HLECs after co‐culturing with SCC‐9‐exos derived from SCC‐9 cells overexpressing miR‐128‐3p (Figure 6D).

## 4. Discussion

During tumor development, an inadequate oxygen supply relative to the demands of rapidly proliferating cells fosters a hypoxic tumor microenvironment, a key driver of progression, treatment resistance, and metastasis [22, 23]. As a prevalent solid tumor, OSCC frequently harbors hypoxic regions, which are strongly associated with aggressive phenotypes [24]. Hypoxia orchestrates lymphangiogenesis—a critical conduit for metastasis—by inducing lymphatic endothelial cell (LEC) proliferation, migration, and tube formation [25]. This process is mediated by a complex network of factors, with HIF‐1α serving as the master regulator that transcriptionally activates key lymphangiogenic factors, including VEGF‐C [26]. In alignment with established methodologies [27], we confirmed the successful establishment of a hypoxic model (1% O_2_) in OSCC cells by demonstrating a robust induction of HIF‐1α expression.

Our findings underscore the role of tumor‐derived exosomes as potent mediators of hypoxia‐driven intercellular communication, operating beyond the canonical function of soluble factors. These EVs (50–150 nm) are increasingly recognized for their roles in cancer pathogenesis [28]. Hypoxia not only augments exosome release but also fundamentally reprograms their molecular cargo, thereby influencing recipient cell behavior [29]. Using differential centrifugation—a widely accepted isolation technique [30]—we purified exosomes from OSCC cells and confirmed their identity through standard characterization methods. Crucially, we demonstrated that Hypo‐exos were internalized by HLECs and significantly enhanced their proliferative, migratory, and tube‐forming capacities, surpassing the effects of exosomes from normoxic cells. This positions Hypo‐exos as active contributors to the remodeling of the lymphatic microenvironment, facilitating metastasis.

The functional impact of exosomes is largely dictated by their cargo, particularly miRNAs, which post‐transcriptionally regulate gene expression in recipient cells. Prior evidence, such as the role of exosomal miR‐1468‐5p in cervical cancer [31], underscores the capacity of exosomal miRNAs to modulate LEC biology. To systematically identify miRNAs responsible for the observed pro‐lymphangiogenic phenotype, we performed miRNA sequencing on Tca‐8113‐exos from both hypoxic and normoxic environments, followed by target prediction and a literature review. This led us to identify four miRNAs (miR‐128‐3p, miR‐365b‐3p, miR‐21‐3p, and miR‐132‐3p) potentially involved in lymphangiogenic regulation. RT‐qPCR analysis revealed that miR‐128‐3p was markedly downregulated under hypoxic conditions. Further experiments in HLECs showed that overexpression of miR‐128‐3p could partially reverse the pro‐lymphangiogenic effects induced by hypoxic SCC‐9‐exos, impacting cell proliferation, migration, and tube formation.

We sought to elucidate the molecular mechanism underlying miR‐128‐3p’s function, a process often regulated by exosomal molecules [32]. Bioinformatics analysis nominated VEGF‐C, a well‐established central driver of lymphangiogenesis that signals through VEGFR3 on LECs to promote their growth and motility [33], as a top predicted target of miR‐128‐3p. Our experimental data robustly support this prediction: western blot analysis confirmed that miR‐128‐3p overexpression in exosome‐donor cells led to reduced VEGF‐C protein levels in recipient HLECs. Most definitively, dual‐luciferase reporter assays demonstrated that miR‐128‐3p directly binds to the 3^′^UTR of VEGF‐C. Thus, we identify a direct mechanistic link whereby the loss of exosomal miR‐128‐3p derepresses VEGF‐C expression in HLECs, thereby stimulating lymphangiogenesis.

The observed suppression of HLEC proliferation by exosomal miR‐128‐3p warrants discussion regarding its underlying cellular mechanism. While this study did not include specific assays for apoptosis or cell cycle distribution, the phenotypic and molecular evidence points towards a likely mechanism. The anti‐proliferative effect, as measured over time in CCK‐8 and colony formation assays, is consistent with a reduction in cell cycle progression rather than acute cell death. This is mechanistically aligned with the suppression of VEGF‐C, a central mitogenic factor for LECs that promotes cell cycle entry and progression primarily through the PI3K/Akt and MAPK pathways. Therefore, it is highly plausible that the downregulation of VEGF‐C by miR‐128‐3p induces a cell cycle arrest in HLECs. Future studies specifically designed to analyze cell cycle phases and apoptotic markers will be invaluable to confirm this inference and pinpoint the precise checkpoint involved.

Our findings that exosomal miR‐128‐3p acts as an inhibitor of lymphangiogenesis appear to contrast with reports demonstrating its pro‐angiogenic role in other contexts [34, 35]. This dichotomy underscores the profoundly context‐dependent nature of miRNA biology. The net effect of a miRNA is determined by the specific target genes it regulates in a given cell type and physiological setting. While miR‐128‐3p may target pro‐angiogenic factors in blood endothelial cells, our data unequivocally demonstrate that in LECs, it directly targets and suppresses VEGF‐C—a master regulator of lymphangiogenesis. Furthermore, the unique hypoxic tumor microenvironment of OSCC, which reprograms exosomal cargo and recipient cell responsiveness, likely shapes this distinct functional outcome. Therefore, our study reveals a novel, context‐specific role of miR‐128‐3p in regulating lymphatic vessel formation, which is distinct from its functions in angiogenesis.

Our study mechanistically bridges a critical gap in our understanding of how the hypoxic tumor microenvironment promotes lymphangiogenesis in OSCC. While hypoxia is known to upregulate VEGF‐C transcriptionally, our data reveal an additional, post‐transcriptional layer of regulation mediated by exosomes. We propose that the hypoxia‐induced downregulation of exosomal miR‐128‐3p serves as a key switch that derepresses VEGF‐C protein synthesis in recipient LECs, thereby amplifying the pro‐lymphangiogenic signal. The consistency of these results across two OSCC cell lines (Tca‐8113 and SCC‐9) underscores the generalizability of this pathway. While miR‐128‐3p is established as a key mediator, the broader miRNA landscape within Hypo‐exos—potentially involving synergistic actions with other differentially expressed species such as miR‐21‐3p—likely contributes to the observed phenotype and warrants further investigation. In addition, the role of exosomal proteins in modulating the tumor microenvironment merits future investigation. Overall, our work not only delineates a miR‐128‐3p/VEGF‐C axis in OSCC lymphangiogenesis but also underscores the therapeutic promise of targeting exosomal miRNA cargo to suppress metastatic dissemination.

This study has several limitations that should be acknowledged. First, its conclusions are derived entirely from in vitro models, and the lack of in vivo validation remains a constraint. Future studies employing xenograft models are needed to confirm the physiological relevance of our findings. Second, our functional assays lacked a consistent “no‐exosome” control group, which limits the precise quantification of the absolute effect magnitude induced by the exosomes themselves. However, the primary conclusions of this study, which are based on the consistent and significant differences observed between hypoxic and Norm‐exos, remain fully supported. Third, for the exosome uptake assay, while rigorous washing steps were included to remove unincorporated dye, the lack of a formal dye‐only control must be noted. Future work will incorporate this critical control to further solidify these findings. Fourth, the use of synthetic miRNA mimics for gain‐of‐function studies, while standard, can lead to supraphysiological miRNA levels that carry a risk of off‐target effects. Although our conclusion of the miR‐128‐3p/VEGF‐C axis is strongly supported by the direct target validation via luciferase assay, the utilization of more precise genetic tools in the future would help to rule out any potential non‐specific effects. Fifth, the functional assays were conducted using exosomes isolated by differential ultracentrifugation alone. While this method is widely accepted and our key cargo finding (miR‐128‐3p downregulation) was consistent, we cannot fully rule out a potential contributory role of minor co‐purified components in the observed phenotypes. Future studies utilizing exosomes purified by additional techniques for functional experiments would provide further refinement. Sixth, while we provide a reasoned argument that the anti‐proliferative effect of miR‐128‐3p is likely due to cell cycle arrest subsequent to VEGF‐C suppression, definitive experiments such as flow cytometry for cell cycle and apoptosis analysis were not conducted. Elucidating the precise cellular mechanism remains an important goal for future work. Seventh, our analysis focused on miRNAs common to both Norm‐exos and hypoxic exosomes; investigating hypoxia‐specific exosomal miRNAs represents an important future direction. Eighth, and most critically from a translational perspective, is the absence of clinical validation. A pivotal next step will be to measure miR‐128‐3p levels in plasma‐derived exosomes and matched tumor tissues from well‐annotated OSCC cohorts, and to correlate its expression with clinicopathological parameters, especially lymph node metastasis and patient survival. Finally, while our mechanistic investigation centered on miR‐128‐3p, the role of other differentially expressed miRNAs, particularly the markedly upregulated oncomiR miR‐21‐3p, warrants further investigation to elucidate the broader regulatory network governing cancer progression.

## 5. Conclusions

Exosomes derived from hypoxia‐induced OSCC significantly enhance the proliferation, migration, and tubulogenesis capabilities of HLECs. Our findings demonstrate that exosomal miR‐128‐3p functions as a key inhibitor of lymphangiogenesis by targeting VEGF‐C, suggesting that therapeutic strategies aimed at restoring miR‐128‐3p expression may hold promise for inhibiting lymphangiogenesis and metastasis in OSCC.

**Nomenclature**
OSCC: Oral squamous cell carcinoma
HLECs: Human lymphatic endothelial cells
HIF‐1α: Hypoxia‐inducible factor 1‐alpha
Hypo‐exos: Hypoxic OSCC cell‐derived exosomes
Norm‐exos: Normoxic OSCC cell‐derived exosomes
LNM: Lymph node metastasis
VEGF: Vascular endothelial growth factor
FBS: Fetal bovine serum
TEM: Transmission electron microscope
NTA: Nanoparticle tracking analysis
WT: Wild type
MUT: Mutant type.

## Author Contributions

Xiaohua Hu, Furong Mao, and Kesheng Zhang conceived the project. Tengchao You, Furong Mao, and Kesheng Zhang designed the experiments. Tengchao You, Furong Mao, Kesheng Zhang, and Xulin Zhang conducted all the experiments and analyzed the data. Tengchao You wrote the manuscript. Xiaohua Hu reviewed and polished the manuscript.

## Funding

This work was supported by grants from the Science and Technology Plan Project of Guizhou Province (Qiankehe Foundation–ZK [2023] General 537) and Zunyi City Science and Technology Project (ZSKH HZ Nos. (2023) 87, (2024) 341 and (2025) 295).

## Ethics Statement

This study used four established public databases, all of which provide deidentified, open‐source data. As the research did not involve animal or human subjects, no ethical approval was required.

## Conflicts of Interest

The authors declare no conflicts of interest.

## Data Availability

The data that support the findings of this study are openly available in miRTarBase, miRDB, TargetScan, and miRBase at https://mirtarbase.cuhk.edu.cn/, https://mirdb.org/, https://www.targetscan.org/vert_80/ and https://www.mirbase.org/.
